# Connectivity-based neurofeedback: Dynamic causal modeling for real-time fMRI^☆^

**DOI:** 10.1016/j.neuroimage.2013.05.010

**Published:** 2013-11-01

**Authors:** Yury Koush, Maria Joao Rosa, Fabien Robineau, Klaartje Heinen, Sebastian W. Rieger, Nikolaus Weiskopf, Patrik Vuilleumier, Dimitri Van De Ville, Frank Scharnowski

**Affiliations:** a Department of Radiology and Medical Informatics, University of Geneva, Geneva, Switzerland; b Institute of Bioengineering, Ecole Polytechnique Fédérale de Lausanne (EPFL), Lausanne, Switzerland; c Computer Science Department, University College London, London, UK; d Department of Neuroscience, CMU, University of Geneva, Geneva, Switzerland; e Geneva Neuroscience Center, Geneva, Switzerland; f Institute of Cognitive Neuroscience, University College London, London, UK; g Swiss Center for Affective Sciences, University of Geneva, Geneva, Switzerland; h Wellcome Trust Centre for Neuroimaging, UCL Institute of Neurology, University College London, London, UK

**Keywords:** Functional magnetic resonance imaging (fMRI), Real-time fMRI, Neurofeedback, Brain connectivity, Dynamic causal modeling (DCM)

## Abstract

Neurofeedback based on real-time fMRI is an emerging technique that can be used to train voluntary control of brain activity. Such brain training has been shown to lead to behavioral effects that are specific to the functional role of the targeted brain area. However, real-time fMRI-based neurofeedback so far was limited to mainly training localized brain activity within a region of interest. Here, we overcome this limitation by presenting near real-time dynamic causal modeling in order to provide feedback information based on connectivity between brain areas rather than activity within a single brain area. Using a visual–spatial attention paradigm, we show that participants can voluntarily control a feedback signal that is based on the Bayesian model comparison between two predefined model alternatives, i.e. the connectivity between left visual cortex and left parietal cortex vs. the connectivity between right visual cortex and right parietal cortex. Our new approach thus allows for training voluntary control over specific functional brain networks. Because most mental functions and most neurological disorders are associated with network activity rather than with activity in a single brain region, this novel approach is an important methodological innovation in order to more directly target functionally relevant brain networks.

## Highlights

• We adapt DCM for use in neurofeedback experiments.
• Participants can control a DCM-based neurofeedback signal.
• Real-time DCM allows for voluntary control over brain connectivity.

## Introduction

Neurofeedback is a technique that provides moment-to-moment information about the current level of brain activity, to which we otherwise do not have conscious access to. Such information can be used to learn voluntary self-regulation of brain activity. Until recently, neurofeedback was mainly used to train self-regulation of autonomic bodily functions (Kimmel, 1974; Miller, 1969; Shearn, 1962), or of specific electroencephalography (EEG) components, for example, in order to communicate with severely paralyzed patients (Birbaumer et al., 1999; Kübler et al., 2001), to suppress epileptic activity (Kotchoubey et al., 2001), or to treat symptoms of attention deficit hyperactivity disorder (Fuchs et al., 2003). However, neurofeedback with EEG is limited with respect to spatial specificity and thus the choice of brain regions that can be targeted.

Recent technological advances in the field of functional magnetic resonance imaging (fMRI) have now made it possible to analyze the data in real-time and thus to provide neurofeedback based on real-time fMRI (rtfMRI). Neurofeedback based on rtfMRI offers the advantage of targeting spatially localized activity in the range of millimeters across the entire brain (deCharms, 2007, 2008; Weiskopf et al., 2004, 2007). Several studies have applied this technically challenging method and demonstrated the feasibility of self-regulating activation in specific brain areas (Bray et al., 2007; Caria et al., 2007, 2010; Chiew et al., 2012; deCharms et al., 2004, 2005; Haller et al., 2010; Hamilton et al., 2011; Hampson et al., 2011; Johnson et al., 2012; Johnston et al., 2010, 2011; Koush et al., 2012; LaConte et al., 2007; Mathiak et al., 2010; Rota et al., 2009; Scharnowski et al., 2012; Shibata et al., 2011; Subramanian et al., 2011; Veit et al., 2012; Weiskopf et al., 2003; Yoo et al., 2007, 2008). The brain regions targeted by these studies are involved in a variety of functions such as perception, motor control, linguistics, and emotional control. Some studies have shown that self-regulation leads to behavioral effects that are specific to the functional role of the targeted cortical area, e.g. improved visual perception due to self-regulation of visual cortex activity (Scharnowski et al., 2012; Shibata et al., 2011), or faster motor responses due to neurofeedback training of the primary motor cortex (Bray et al., 2007); see also (Caria et al., 2007; deCharms et al., 2005; Rota et al., 2009; Weiskopf et al., 2003, 2004). A few studies even demonstrated therapeutic effects of rtfMRI-based neurofeedback training in chronic pain patients (deCharms et al., 2005), Parkinson's disease (Subramanian et al., 2011), tinnitus (Haller et al., 2010), and depression (Linden et al., 2012). Further, it has been shown that learning voluntary control over activity within an ROI indirectly induces also network changes (Lee et al., 2011, 2012; Rota et al., 2011; Scharnowski et al., 2012; Zotev et al., 2011).

Apart from a few studies that targeted multivariate patterns of brain activity (Chiew et al., 2012; LaConte et al., 2007; Shibata et al., 2011), all of the above mentioned rtfMRI-based neurofeedback studies trained participants to control localized brain activity within a region of interest (ROI). However, although neuroimaging studies have firmly established functional specialization as a principle of brain organization, most mental functions and most neurological conditions are associated with functional integration of interconnected networks (Bullmore, 2012; Desseilles et al., 2011; Lynall et al., 2010; Seghier et al., 2010). Such integration between different brain areas has proven more difficult to assess, but recent developments in data analysis techniques now make the study of such functional connectivity feasible (Friston et al., 2003; McKeown et al., 1998; Roebroeck et al., 2005). Two fundamental approaches to study brain connectivity can be distinguished: data-driven approaches and hypothesis-driven approaches. The former is purely explorative and does not require a priori hypotheses about the functional network. For example, independent component analysis (ICA) can be used to decompose the fMRI data into a set of functionally relevant maps without having to define ROIs a priori (Calhoun et al., 2001; Damoiseaux et al., 2006; Esposito et al., 2003; Greicius et al., 2003; McKeown et al., 1998; Xie et al., 2011). On the other hand, the hypothesis-driven approach makes use of prior knowledge about the underlying network and requires a model describing how neural dynamics propagates through a set of interconnected ROIs.

A particularly influential hypothesis-driven approach in fMRI is dynamic causal modeling (DCM) (Friston et al., 2003, 2007; Kiebel et al., 2007; Stephan et al., 2007a, 2010). DCM requires defining hypotheses about the neural mechanisms underlying a specific fMRI measurement in terms of ROIs, connections between these ROIs, external inputs to the network (e.g., visual stimulation), and context dependent manipulations of the network (e.g., attention). Using Bayesian model comparison, DCM allows for comparing which model architecture explains the data best (Penny et al., 2004). DCM also allows for estimating the individual model parameters and thus, for example, shedding light on the dynamic connectivity changes during an experiment.

In the present study we adapted DCM for use in neurofeedback experiments. Near real-time DCM (rtDCM) was accomplished by (1) optimizing the trade-off between model convergence precision and computational speed, by (2) integrating rtDCM into the real-time analysis pipeline with rigorous pre-processing of the ROI time courses (Koush et al., 2012), and by (3) generating a feedback signal from the results of a Bayesian model comparison between two predefined model alternatives. In contrast to conventional offline DCM, where one seeks to find the model that explains the data best, our approach requires the participants to modulate the data (i.e. effective connectivity) so that one of two predefined models dominates over the other. Our approach thus allows for training a very specific model with a pre-defined connectivity architecture.

Here, in a proof-of-concept experiment, we asked participants to voluntarily modulate the connectivity either between left visual cortex and left parietal cortex, or between right visual cortex and right parietal cortex. Modulation of connectivity was accomplished by shifting visual–spatial attention either to the left or to the right visual field. During shifts of attention to the left/right, connectivity between the right/left visual and parietal cortices will be increased. The connectivity between visual and parietal areas has been studied intensely and can be voluntarily modulated by visual–spatial attention (Blankenburg et al., 2010; Bressler et al., 2008; Greenberg et al., 2010; Hopfinger et al., 2000; Kastner et al., 1999; Kelley et al., 2008; Lauritzen et al., 2009; Ruff et al., 2006, 2009; Silver et al., 2005, 2007; Vossel et al., 2012; Yantis et al., 2002); it is thus a well suited paradigm to explore the feasibility of near real-time connectivity feedback based on DCM in a proof-of-concept study. We hypothesized that (a) the connectivity difference due to attentional modulation can be determined in near real-time using rtDCM, and that (b) the participants will be able to control the connectivity-based feedback signal.

Our new approach goes beyond previous neurofeedback studies that only provided feedback from activity in specific ROIs. By adapting state-of-the-art connectivity measures such as DCM for neurofeedback, it is now possible to learn voluntary control over functional brain networks. Because most mental functions and most neurological disorders are associated with network activity rather than with activity in single ROIs, this novel approach is an important methodological innovation in order to more specifically target such brain networks.

## Material and methods

### Data acquisition

MRI data were acquired on a Siemens 3 T Trio scanner (Siemens Healthcare, Erlangen, Germany) at the Brain and Behavior Laboratory (University of Geneva). Functional images were obtained with a single-shot gradient-echo T2*-weighted EPI sequence (TR = 1000 ms, 16 slices volumes, matrix size 64 × 64, voxel size = 3 × 3 × 3.75 mm^3^, flip angle α = 77°, bandwidth 2.23 kHz/pixel, TE = 30 ms), using a 12-channel phased array coil. The first 10 EPI volumes were discarded to account for T1 saturation effects. At the beginning of the scanning session, a T1-weighted structural image was acquired for every participant (MPRAGE, 1 mm isotopic resolution). Heart rate, respiration, and eye movements were continuously monitored throughout the experiment with a modular data acquisition system (MP150, 1 kHz sampling rate, BIOPAC Systems Inc.) and with an infrared eye-tracking system (ASL 450, 60 Hz sampling rate, LRO System), respectively. Heart rate was measured using a pulse oximetry sensor and respiration was measured using an elastic belt around the participant's chest. Visual stimuli and instructions were displayed using a rectangular projection screen at the rear of the scanner bore with a mirror positioned within the head-coil.

### Participants

Fourteen healthy human volunteers (5 male, 9 female, age 27.2 ± 5.2 years) with normal or corrected-to normal vision gave written informed consent to participate in the experiment, which was approved by the local ethics committee. Participants were naïve to neurofeedback and were paid 25 CHF/h for their participation. Six pilot participants (2 male, 4 female, age 26.5 ± 7.1 years) performed only the functional localizer runs. Data from these localizer runs was used to optimize the rtDCM pipeline. Seven participants (3 male, 4 female, age 27.7 ± 3.3 years) performed the complete experiment. One of the participants had to be excluded from the analysis because of poor compliance. Before the experiment, participants received instructions that they will attempt to control their connectivity between brain areas and receive neurofeedback information about their success. The instructions included an explanation of the task conditions as well as the neurofeedback display, and recommended the use of shifting visual-spatial attention as a potential regulation strategy. However, it was emphasized that participants should find an individual strategy that worked best. It was also explained to the participant, that they will receive an additional reward of 1 CHF for each successful neurofeedback trial. Further, they were instructed to fixate on the central fixation point throughout the experiment, to breathe steadily, and to remain as still as possible.

### Functional localizer runs

Before the neurofeedback runs, we ran a functional localizer that consisted of three successive runs to delineate each participant's left and right early visual cortex (VC), and the left and right superior parietal lobule (SPL), respectively. The first localizer run delineated the early visual cortex of the left and right hemisphere. It consisted of 11 baseline blocks interleaved with 10 blocks of flickering circular checkerboards. All blocks were 10 s long. The checkerboards were presented simultaneously in the left and right visual field (diameter of 5° visual angle; eccentricity 5° visual angle; 100% contrast, 8 Hz contrast reversal).

The second and third localizer runs delineated the right and left SPL, respectively. Each SPL localizer run consisted of 11 baseline blocks interleaved with 10 blocks of shifting attention covertly (i.e. without moving their eyes) to the left or right visual field. Changes in the fixation point instructed participants to shift attention to the left or right visual field. The target location for shifting attention was illustrated by low-contrast dashed circles, which were presented at the same location as the visual checkerboards during the VC localizer run. The total duration of the visual and the attention localizer runs was 10.3 min.

**Fig. 1:**
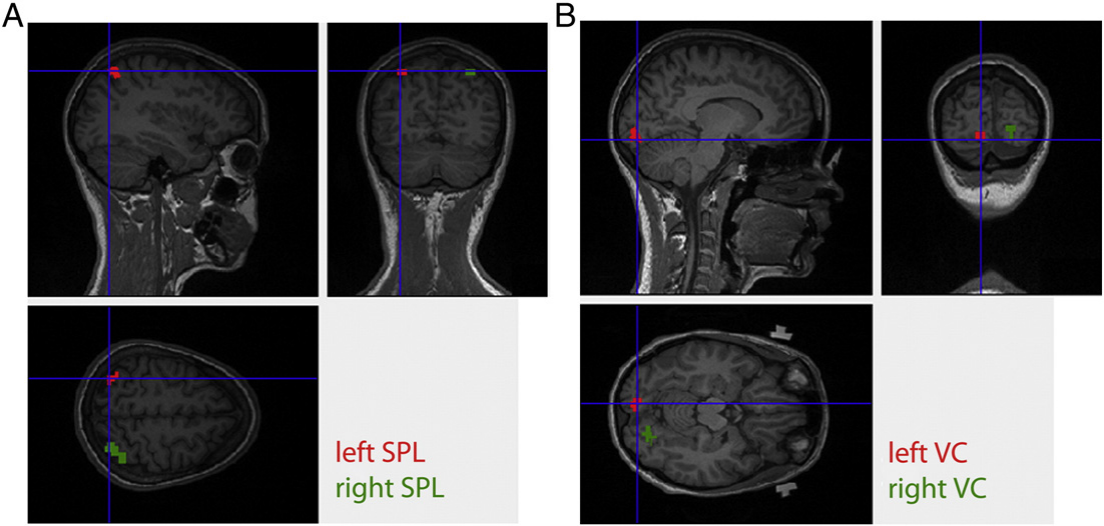
Illustration of the ROIs. Left and right (A) SPL and (B) VC ROIs of a representative participant are shown on sagittal, transverse and coronal planes of this participant's structural scan. The left/right SPL ROI enclosed on average 24.4 ± 12.6 voxels, and the left/right VC 16.3 ± 8.2 ROI voxels. SPL — superior parietal lobule, VC — visual cortex.

Immediately after acquiring the localizer runs, the images were pre-processed with SPM8 (Wellcome Trust Centre for Neuroimaging, Queen Square, London, UK), i.e. they were corrected for slice time acquisition differences, realigned to the first scan of each run, smoothed with an isotropic Gaussian kernel with 4 mm full-width-at-half-maximum (FWHM), and coregistered to the structural scan. Next, we specified a general linear model (GLM) with regressors for the experimental conditions. Using the MarsBar toolbox (Brett et al., 2002), the ROIs were then defined as those voxels in left/right occipital cortex and left/right SPL that exhibited a significant positive BOLD response to the visual stimulation or the shifts of attention, respectively (Fig. 1; p < 0.05 corrected for multiple comparisons using FWE).

### Neurofeedback runs

**Fig. 2:**
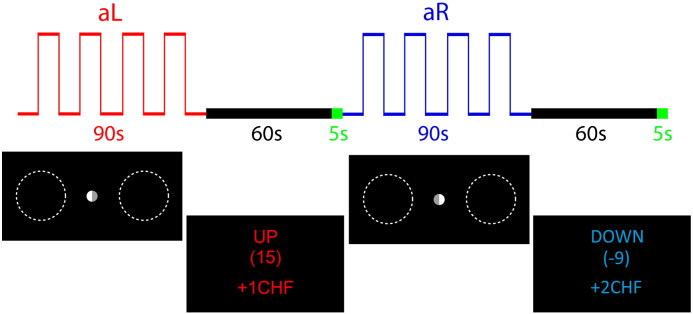
Neurofeedback trials. Shown is an aL trial followed by an aR trial. Each individual trial consisted of 5 baseline blocks interleaved with 4 blocks of shifting attention to the left for aL (red curve) and to the right for aR trials (blue curve). Following 60 s of resting state acquisition during which the neurofeedback value was computed (black timeline), the feedback signal was displayed to the participant for 5 s (green timeline). The feedback display indicated the condition (UP in red for aL; DOWN in blue for aR), the rounded logarithmic Bayes factor, and the cumulative reward. For illustration purposes, the fixation point is enlarged and the dashed circles are of higher contrast.

In three subsequent neurofeedback runs, we then tested the ability of participants to voluntarily control the feedback signal by covertly shifting their visual–spatial attention. Each of the neurofeedback runs consisted of 8 neurofeedback trials. Each neurofeedback trial consisted of 5 baseline blocks interleaved with either 4 blocks of attention to the left (aL) or to the right (aR) (Fig. 2; all blocks were 10 s; attention left/right conditions alternated).

The attention conditions were indicated by changes in the fixation point, i.e. participants were informed whether attention to the left or attention to the right will be most effective in order to control the feedback signal. The target location for shifting attention was illustrated by low-contrast dashed circles. They were present on both sides during all trials. No other visual stimuli were presented. Each neurofeedback trial was followed by a 60 s block of resting state acquisition (during this time the feedback signal was computed) and a 5 s block during which the feedback signal was presented to the participant. During baseline blocks, participants were instructed to count backwards. During the resting state blocks, participants were asked to close their eyes. After the resting state block, 3 auditory beeps and 3 visual flashes indicated to the participant that the feedback and reward values were displayed on the screen. The feedback display signal consisted of either the word UP (in red), which indicated aL trials, or DOWN (in blue), which indicated aR trials, the rounded logarithmic Bayes factor value in brackets (which in case of success was positive for aL trials and negative for aR trials), and the total reward that had been earned until the present trial. Details about the logarithmic Bayes factor values are provided below.

### Real-time data processing and computation of the connectivity-based neurofeedback signal

Immediately after the acquisition of an fMRI image, the image was exported to a standard local PC (CPU Intel Core i7-2620 M 2.7 GHz, 4 GB RAM) and processed with custom-made real-time fMRI software (Koush et al., 2012) running on MATLAB (Mathworks Inc., Natick, MA, USA). This software was used to perform online motion correction, to extract the time courses from the ROI masks, to remove signal drift, spikes, as well as high frequency noise, and to calculate the feedback signal (for details see Koush et al., 2012; the toolbox with the rtDCM extension is available on request from the corresponding author).

The connectivity-based neurofeedback signal was calculated by adapting DCM10 (as implemented in SPM8) for near real-time purposes. DCM is a mathematical framework for modeling effective connectivity between interacting brain regions at the neuronal level as a set of coupled differential equations (Friston et al., 2003; Stephan et al., 2007a, 2010). These equations describe how the putative neuronal population activity in ROIs changes over time, depending on the connectivity architecture of the model, and on experimentally controlled inputs. The inputs can be direct input acting on specific ROIs (e.g. sensory stimulation), or they can be contextual modulations of the connections between ROIs (e.g. attention). Using a hemodynamic forward model, the neuronal dynamics is translated into BOLD signal (Buxton et al., 1998; Friston et al., 2000; Stephan et al., 2007b), and the a posteriori parameters of the model can be estimated within a Bayesian framework (Friston, 2002; Friston et al., 2007). In order to compare between multiple hypothetical brain network models, Bayesian model comparison can be used to determine the most likely model given the measured BOLD signal (Penny et al., 2004; Stephan et al., 2009).

**Fig. 3:**
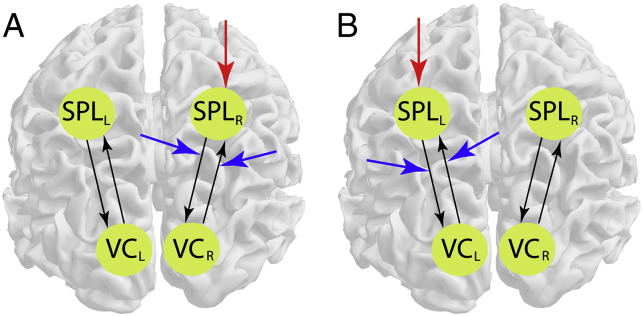
Models representing (A) attention to the left and (B) attention to the right. (A) Covert shifts of visual–spatial attention to the left modulate the right SPL, and the connectivity between the right SPL and the right VC. (B) Covert shifts of visual–spatial attention to the right modulate the left SPL, and the connectivity between the left SPL and the left VC. Red and blue arrows represent the external and modulatory inputs of attention, respectively. SPL — superior parietal lobule, VC — visual cortex, L — left hemisphere, R — right hemisphere.

Adapting DCM for near real-time purposes involved optimizing the trade-off between DCM model convergence precision and computational speed, by integrating DCM into the real-time pipeline, and by generating a feedback signal from the result of a Bayesian model comparison between two model alternatives. The two models that we compared represented covert shifts of visual–spatial attention to the left or right visual field, i.e. they consisted of 4 ROIs, which are the interconnected left visual and parietal cortices and the interconnected right visual and parietal cortices (Fig. 3).

What differed between the models was the external and the modulatory inputs of attention, which should be stronger on the right SPL and on the connectivity between the right VC and the right SPL when attention is covertly shifted to the left visual field (Fig. 3A, model M_aL_), and stronger on the left SPL and on the connectivity between left VC and the left SPL when attention is covertly shifted to the right visual field (Fig. 3B, model M_aR_).

The model comparison resulted in a logarithmic Bayes factor, which indicated the relative dominance of one model over the other. This logarithmic Bayes factor was presented to the participants as the feedback signal. Dominance of M_aL_ was indicated by positive logarithmic Bayes factors, dominance of M_aR_ was indicated by negative logarithmic Bayes factors. In order to pool results across aL and aR conditions, the logarithmic Bayes factors for the aR conditions were inverted, i.e. in the Results section, positive logarithmic Bayes factors indicate the dominance of the correct model and hence successful control over the feedback signal.

### Optimization of near real-time DCM

Initially, the optimization of the near real-time DCM approach was based on the SPL localizer runs of the 6 pilot participants. This initial optimization was used to define the parameters for the neurofeedback runs of the 7 participants who were scanned subsequently. After the neurofeedback experiment, the optimization was extended to now include the localizer runs of all 13 participants. In order to find the optimal neurofeedback trial length that allows a fast and stable model comparison performance, we ran our algorithm for different sliding window lengths of 30, 50, 70, 90, 110, 130, 150, 170, 190, and 210 scans (the latter constitutes the complete time course). Each of the sliding windows was shifted across the respective functional localizer time course in steps of 20 time points. Please note that the sliding window length limited the number of possible shifts across the functional localizer time course. For each sliding window length, we evaluated how often the near real-time Bayesian model comparison (i.e. the logarithmic Bayes factor computed for two compared models) reflected the respective experimental condition, i.e. how often M_aL_ was dominant during aL conditions and vice versa for M_aR_ and the aR conditions. Statistically, the performance of each sliding window was evaluated by a non-parametric sign-statistics (one-tailed, median > 0). We also evaluated for each sliding window length, how many iterations were required until the Bayesian model comparison algorithm converged. Further, we evaluated for each sliding window length the time it took to compute a single iteration of a single DCM model estimation algorithm. Based on the results from this analysis and in order to ensure that the Bayesian model comparison could be completed within the 60 s resting state blocks, we limited the single model estimation algorithm during the neurofeedback experiment to 44 iterations. Near real-time DCM model estimation was performed based on SPM8 DCM10 module functions.

### Offline analyses

In order to evaluate voluntary control over the feedback signal, we quantified the exceedance probabilities (Stephan et al., 2009; Pe) in favor of M_aL_ (*Pe_MaL_*) and M_aR_ (*Pe_MaR_*) for the aL and aR conditions separately using random effect Bayesian model selection (RFX BMS, SPM8 DCM10 module). In addition, we analyzed the logarithmic Bayed factors by calculating the median (*m*), the interquartile range (*iqr*), and non-parametric sign test statistics (one-tailed, median > 0). Z-statistics was used to approximate the p-values of the non-parametric sign test. This approach was used because a Jarque–Bera test established that the logarithmic Bayes factors were not normally distributed.

**Fig. S1:**
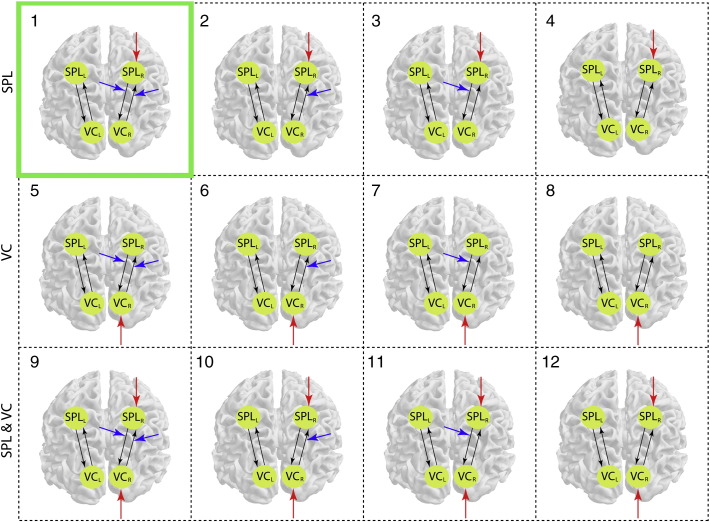
Alternative DCM models used for performance comparison. The models received direct input of attention either on the SPL (1st row), the VC (2nd row), or both (3rd row). Within each of these model families, attention could either modulate top-down as well as bottom-up connections (1st column), only top-down connections (2nd column), only bottom-up connections (3rd column), or none of them (4th column). The model that was used in the neurofeedback runs was model 1 (highlighted green).

In order to compare the performance of the specific model architecture that was used in our experiment with other models, we post-hoc evaluated the feedback signal (i.e. the logarithmic Bayes factor) for a subset of 11 plausible models. These models either had direct input of attention into the SPL, into the VC, or into both (Inline Supplementary Fig. S1). For any of these three model families, all possible combinations of modulatory inputs of attention on the connections were evaluated (top-down and bottom-up, top-down only, bottom-up only, none). Because we compared performance for the attention localizer run and for the neurofeedback runs, this analysis was based on data from the seven participants who performed both, i.e. the localizer data from the 6 pilot participants who did not perform the neurofeedback runs were not used in this analysis. As a performance measure, we compared successful and failed trials by calculating the z-statistics of a non-parametric sign test.

Inline Supplementary Figure S1

We also compared the new connectivity-based feedback signal to classical activity-based differential feedback signals. For this, we calculated for each neurofeedback trial the difference between the left and right VC and SPL percent signal changes, respectively. The same statistical analysis as for the connectivity-based feedback was applied to the activity-based results.

## Results

### Optimization of near real-time DCM

In order to achieve a fast and stable Bayesian model comparison performance, we first optimized the length of the sliding window over which the data were analyzed, and thus the length of a single neurofeedback trial. We found that a sliding window of 90 scans was sufficient to reliably determine the dominant model (i.e. M_aL_ vs. M_aR_; Table 1). In our experiment, such a sliding window corresponded to five 10 s baseline blocks interleaved with four 10 s blocks of self-regulation. For large sliding windows of 190 and 210 scans (which corresponded to the complete localizer run and thus to a standard offline DCM analysis), the model comparison performance decreased.

**Fig. S2:**
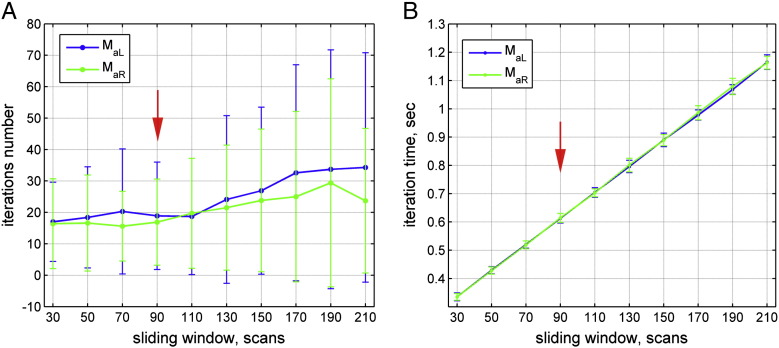
Computational speed of the model estimation algorithm. (A) The number of iterations until the model estimation algorithm converged remained low and stable for sliding window lengths of less than 110 scans. For longer sliding window lengths, the number of iterations increased and became less predictable. (B) The computational time each iteration took depended linearly on the length of the sliding window. For the neurofeedback runs, we used a sliding window of 90 scans (red arrows). The green and blue lines represent estimation of the M_aL_ and M_aR_ models, respectively. The black/blue error bars represent the standard deviations.

Besides the length of a neurofeedback trial, the number of iterations of the model estimation algorithm until it converged and the computational time per iteration were further parameters that we optimized. We found that the number of iterations remained at around 20 ± 20 for sliding window lengths of less than 110 scans (Inline Supplementary Fig. S2A). For longer sliding window lengths, the number of iterations and especially their variance increased. This might lead to delays in computing the feedback signal because more iterations might be required. The computational time per iteration increased linearly for increasing sliding window lengths (Inline Supplementary Fig. S2B). For the sliding window lengths comprising 90 scans, which we used for the neurofeedback experiment, a single iteration on a standard PC took 0.61 ± 0.016 s.

Inline Supplementary Figure S2

In our neurofeedback experiment, we limited the number of iterations to 44 for a single model estimation. This threshold was reached in 16.7% of all neurofeedback trials, meaning that only a minority of trials were estimated sub-optimally. However, the premature termination of the algorithm only affected the precision of the resulting logarithmic Bayes factor but not its sign, i.e. the classification into correct/incorrect trial and thus the feedback reward was not affected.

### Voluntary control over the connectivity-based neurofeedback signal

**Fig. 4:**
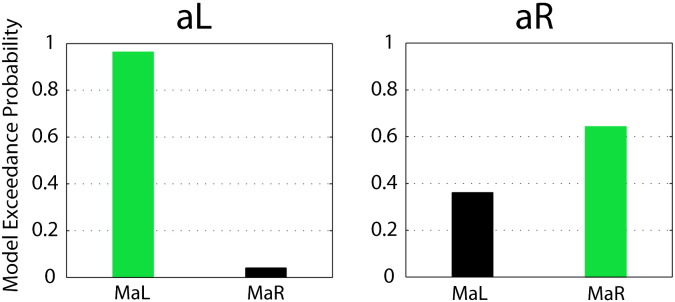
Voluntary control over the feedback signal. Successful control is reflected by increased model exceedance probabilities of M_aL_ during aL and M_aR_ during aR conditions. For each condition, the dominant model is indicated in green.

Participants in our experiment were able to control the connectivity-based feedback signal. Specifically, the exceedance probability of M_aL_ during aL was higher than those of M_aR_ and vice versa during aR (Fig. 4; aL: Pe_MaL_ = 0.97, Pe_MaR_ = 0.03; aR: Pe_MaL_ = 0.36, Pe_MaR_ = 0.64).

**Fig. S3:**
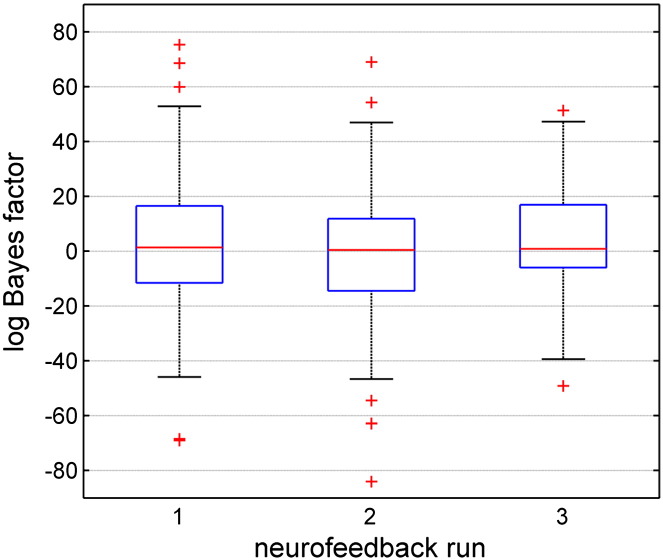
Voluntary control over the feedback signal across neurofeedback runs. Successful control over the feedback signal is reflected by 43 positive feedback signal values, i.e. logarithmic Bayes factors. Control over the feedback signal improved slightly across runs but we did not find robust evidence for neurofeedback learning. The blue box represents 50% of the data, with the lower bound representing the first quartile (*fq*), and the upper bound representing the second quartile (*sq*). The red line represents the median. The black error bars represent the data limits (*fq* − 1.5*iqr*, *sq* + 1.5*iqr*). Red crosses represent trials outside the data limits.

Furthermore, the feedback signal (i.e. the logarithmic Bayes factor) was significantly greater than zero (m = 0.8, fq = − 10.5, iqr = 25.6; one-tailed sign test and z-statistics, sign = 97, z = 1.93, p = 0.027). To investigate if the ability to control the feedback signal changed during the course of the experiment, we evaluated performance in terms of the logarithmic Bayes factors across runs (Inline Supplementary Fig. S3). The feedback signal was greater than zero in the first and the second neurofeedback run, and significantly greater than zero in the third run (one-tailed sign test and z-statistics; m_1_ = 1.3, fq_1_ = − 11.6, iqr_1_ = 28.1, sign_1_ = 32, z_1_ = 0.9, p_1_ = 0.175; m_2_ = 0.4, fq_2_ = − 14.5, iqr_2_ = 26.3, sign_2_ = 29, z_2_ = 0.13, p_2_ = 0.447; m_3_ = 0.85, fq_3_ = − 6.0, iqr_3_ = 22.3, sign_3_ = 36, z_3_ = 2.0, p_3_ = 0.023). Performance improved slightly but non-significantly across neurofeedback runs (non-parametric permutation test of the slope of a linear regression of z-values, N = 999 permutations, p = 0.207; non-parametric permutation test of the slope of linear regression of medians, N = 999 permutations, p = 0.43).

Inline Supplementary Figure S3

**Fig. S4:**
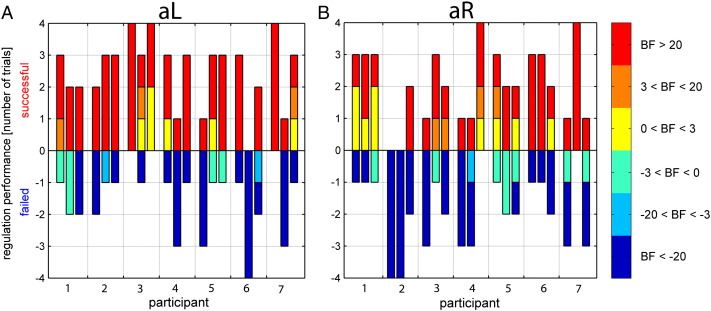
Voluntary control over the feedback signal for each participant across runs, but separately for (A) aL and (B) aR conditions. Participants successfully controlled the feedback signal during aL (A; m = 3.97, fq = − 7.8, iqr = 26.5; one-tailed sign test, z = 2.29, p = 0.01, sign = 53), but not during aR (B; m = 0.12, fq = − 13.6, iqr = 25.6; one-tailed sign test, z = 0.33, p = 0.37, sign = 44). Note that each run consisted of 8 trials. Following standard conventions (Penny et al., 2004), the color represents weak (Bayes factor < 3), positive (3 < Bayes factor < 20), strong and very strong evidence (Bayes factor > 20).

**Fig. 5:**
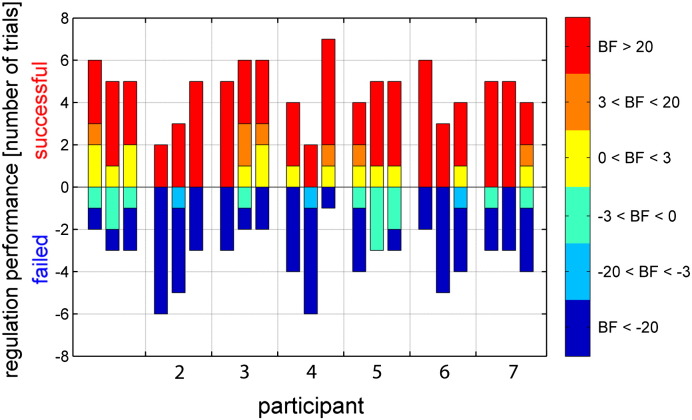
Voluntary control over the feedback signal for each participant across neurofeedback runs. Successful control over the feedback signal is reflected by the number of successful trials per run. Note that each run consisted of 8 trials. Following standard conventions (Penny et al., 2004), the color represents weak (Bayes factor < 3), positive (3 < Bayes factor < 20), strong and very strong evidence (Bayes factor > 20).

To illustrate the individual participant performance, we classified the Bayes factors according to weak (0–3), positive (3–20), strong and very strong evidence (> 20; Fig. 5) (Penny et al., 2004). Whereas performance varied across runs and participants, all but one participant (participant 2) achieved more successful than failed trials. On average, 4.6 ± 1.3 successful and 3.4 ± 1.3 failed trials were observed. As can be expected based on previous behavioral studies that found an attentional bias to the left in healthy participants (Jewell and McCourt, 2000), performance during aL trials is better than performance during aR trials (Supplementary Methods and Inline Supplementary Fig. S4).

Inline Supplementary Figure S4

Voluntary control of the feedback signal was not related to cardio-respiratory artifacts or eye movements, which showed no difference between the experimental conditions in terms of the mean heart rate, mean respiration, as well as mean horizontal and vertical eye-position coordinates; this is also the case for the functional localizer runs (Inline Supplementary Table S1; paired *t*-test; df_loc_ = 12; df_nf_ = 40; heart rate: t_loc_ = − 0.1, p_loc_ = 0.94, t_nf_ = 0.2, p_nf_ = 0.83; respiration, t_loc_ = 0.1, p_loc_ = 0.94, t_nf_ = 0.1, p_nf_ = 0.92; eye movements, t_loc_h_ = 0.2, p_loc_h_ = 0.88, t_loc_v_ = 0.3, p_loc_v_ = 0.75, t_nf_h_ = 0.4, p_nf_h_ = 0.68, t_nf_v_ = 0.4, p_nf_v_ = 0.72).

Inline Supplementary Table S1

**Table S1:** Heart rate [BPM], respiration [a.u.], horizontal and vertical eye movements from the center [° visual angle].

run/ parameter		localizer		neurofeedback	
condition	baseline	condition	baseline
**Heart rate**		66.0±10.8	66.5±10.5	67.6±8.3	67.1±8.0
**Respiration**		60.0±18.3	59.4±18.6	59.3±18.1	58.7±18.4
**Eye movement**	**h**	1.58±0.73	1.51±0.74	1.48±0.71	1.39±0.67
**v**	1.66±0.91	1.52±0.74	1.64±0.91	1.54±0.82

Inline Supplementary Table S1

### Comparison with alternative DCM architectures

**Fig. 6:**
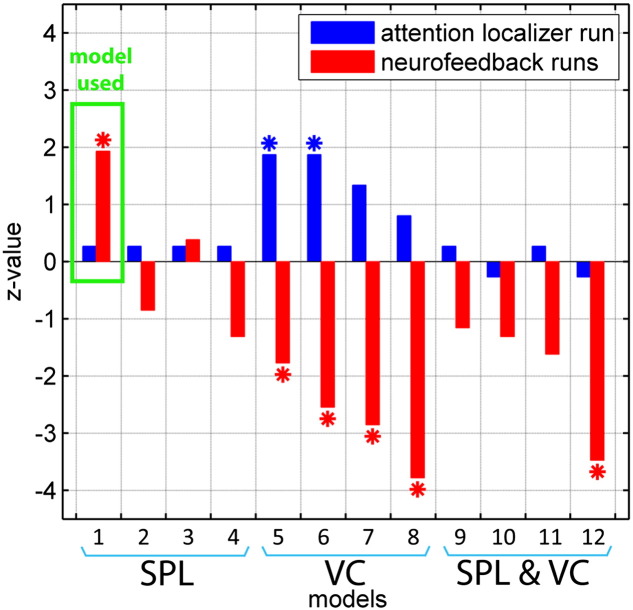
Performance comparison between alternative DCM models. Positive z-values indicate that the respective model architecture reveals control over the feedback signal, i.e. dominance of M_aL_ during aL and dominance of M_aR_ during aR; negative z-values indicate the opposite. During the attention localizer run (blue), the models with direct input of attention into VC worked best (particularly models 5 & 6). During the neurofeedback runs (red), the model with direct input into VC now showed the opposite pattern of results. Interestingly, the model that was used in the neurofeedback runs of our experiment (model 1, highlighted green) performed poorly during the localizer run, but was the only model that performed well during the neurofeedback runs. Details about the model index are provided in Inline Supplementary Fig. S1. Models 1–4 received direct input of attention into the SPL, models 5–8 into the VC, and models 9–12 into the SPL as well as the VC. Asterisks denote statistical significance.

In order to compare the performance of the specific model architecture that was used in our experiment with alternative models, we post-hoc evaluated the feedback signal (i.e. the logarithmic Bayes factor) for a subset of 11 plausible models (see Inline Supplementary Fig. S1 for an overview of the alternative model architectures). We found that during the attention localizer run, models with direct input into VC performed best (Fig. 6). Particularly models 5 & 6 achieved logarithmic Bayes factors that were significantly greater than zero (Fig. 6A, one-tailed sign test and z-statistics, z_model_5_ = 1.9, z_model_6_ = 1.9, p < 0.05). However, during the neurofeedback runs, the models with direct input into VC showed the opposite pattern by indicating dominance of M_aL_ during aR trials and dominance of M_aR_ during aL trials, i.e. they showed logarithmic Bayes factors that were significantly smaller than zero (Fig. 6A, one-tailed sign test and z-statistics, z_model_5_ = − 1.8, z_model_6_ = − 2.6, z_model_7_ = − 2.9, z_model_8_ = − 3.8, z_model_12_ = − 3.5, p < 0.05).

Interestingly, the model that was used during the neurofeedback experiments performed poorly during the attention localizer runs, but performed well during the neurofeedback runs (Fig. 6, model 1; one-tailed sign test and z-statistics, neurofeedback runs: z = 1.93, p = 0.03; Pe_MaL_,_aL_ = 0.97, Pe_MaR_,_aR_ = 0.64; localizer runs: z = 0.27, p = 0.40, Pe_MaL_,_aL_ = 0.72, Pe_MaR_,_aR_ = 0.62). A similarly good performance during the neurofeedback runs was observed for model 3, which also received direct input of attention into SPL as well as top-down modulation of attention (Fig. 6, model 3; z = 0.39, p = 0.35; Pe_MaL_,_aL_ = 0.96, Pe_MaR_,_aR_ = 0.64).

### Comparison with alternative feedback measures

Based on the percent signal changes of the ROI time courses (Table 2), we post-hoc evaluated the performance of alternative feedback measures such as the differential feedback between the left and the right SPL, as well as between the left and right VC. Such inter-hemispheric comparisons reflect the current direction of attention towards the right or the left side of the visual field (Hopfinger et al., 2000; Kastner et al., 1999; Silver et al., 2007).

**Fig. S5:**
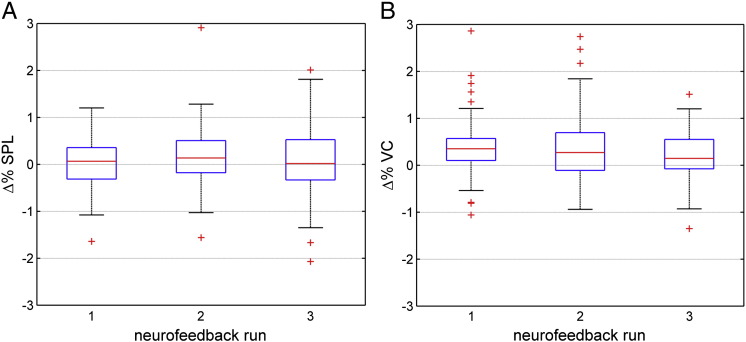
Differential feedback performance across neurofeedback runs. Successful performance of the differential feedback signal is reflected by positive feedback signal values, i.e. percent signal changes between (A) the left and the right SPL, or (B) between the left and the right VC. The differential feedback signal based on SPL activity did not reflect the experimental design, i.e. aL vs. aR. In contrast, the differential feedback signal based on VC activity did reflect the experimental design. The blue box represents 50% of the data, with the lower bound 44 representing the first quartile (*fq*), and the upper bound representing the second quartile (*sq*). The red line represents the median. The black error bars represent the data limits (*fq* − 1.5*iqr*, *sq* + 1.5*iqr*). Red crosses represent trials outside the data limits, i.e. outliers.

Performance based on the differential feedback signal between the left and right SPL was not significant (Inline Supplementary Fig. S5A; one-tailed sign test and z-statistics, sign = 87, z = 0.5, p = 0.32), and did not change significantly across feedback runs (m_1_ = 0.07, m_2_ = 0.14, m_3_ = 0.02; non-parametric permutation test of the slope of linear regression of medians, N = 999 permutations, p = 0.38). The differential feedback signal between the left and the right VC showed a performance that was significantly greater than zero (Inline Supplementary Fig. S5B; one-tailed sign test and z-statistics, sign = 120, z = 5.5, p < 0.001). Interestingly, the performance of the differential VC feedback signal significantly decreased across runs (m_1_ = 0.35, m_2_ = 0.27, m_3_ = 0.15; non-parametric permutation test of the slope of linear regression of medians, N = 999 permutations, p = 0.024).

Inline Supplementary Figure S5

Importantly, there was no correlation between the differential feedback measures and the rtDCM feedback measure (paired F-statistics; differential SPL vs. rtDCM: F < 0.001, p = 0.99; differential VC vs. rtDCM: F = 0.26, p = 0.61). Hence, successful control of the rtDCM feedback does not predict performance based on the VC differential feedback signal, i.e. they appear independent from each other.

## Discussion

In this proof-of-principle study, we showed for the first time that connectivity feedback is possible, and that such a feedback signal can be voluntarily controlled by participants. Our new approach is thus suitable to train voluntary control over functional brain networks. This is an important extension of the neurofeedback approach that allows to directly target brain networks underlying mental functions and neurological disorders.

### Near real-time DCM

Our implementation of effective connectivity feedback was based on rtDCM. DCM is a hypothesis driven approach that requires the formulation of a specific network of connectivity between ROIs and of experimental factors that modulate these connections. Compared to purely explorative approaches to connectivity, DCM makes use of prior knowledge about the connectivity between ROIs and of how such connectivity might be modulated by cognitive factors. This bears the advantage that the hard problem of assessing and training real-time connectivity can be reduced to training the dominance of a very specific pattern of connectivity over another.

Conceptually, our new approach differs from the conventional DCM analysis. With conventional DCM analysis one seeks to find a plausible model that describes the given data best. Near real-time DCM neurofeedback requires the opposite; it requires modulating the data (i.e. brain connectivity) so that a given predefined model dominates. This new approach allows for training a specific model of functional or clinical relevance. In the case of clinical rehabilitation therapy, for example, one can feed back the result of a rtDCM Bayesian model comparison between the model that corresponds to the healthy condition and a model that corresponds to the neurological condition.

To adapt DCM for near real-time purposes, we needed to optimize the trade-off between robustness of the DCM estimation and computational speed. For our specific visual–spatial attention paradigm, a minimum of 90 scans was necessary to achieve a robust estimate (Table 1, Inline Supplementary Fig. S2). The time it took to compute the feedback signal for these 90 scans was less than 1 min. Such a delay seems quite long and is similar to the feedback delays in the early days of activity-based feedback using rtfMRI (Posse et al., 2003; Yoo and Jolesz, 2002; Yoo et al., 2004). However, it is not yet clear how the temporal contiguity affects neurofeedback learning. Intuitively, one might assume that continuous feedback is superior over intermittent feedback, because reinforcement is more directly linked to efforts of the participant, because it provides more opportunities to evaluate training success, and because it keeps the task engagement high. In the field of EEG-based feedback, a few studies indeed reported that a shorter temporal contiguity facilitates learning (Rockstroh et al., 1990; Travis et al., 1974). However, because of the different temporal resolutions of EEG and fMRI, these findings might not be transferable. A recent study, which compared continuous and intermittent neurofeedback based on rtfMRI, found that intermittent presentation of feedback was more effective (Johnson et al., 2012). This might be because of improved signal quality due to more scans being available for averaging, because the intrinsic hemodynamic delay does not have to be taken into account, and because there is no more dual task interference between self-regulation and evaluation of the feedback signal. Hence, although the delay seems long, it remains an empirical question if such a delay precludes or even facilitates efficient neurofeedback training.

We found that performance peaks for a sliding window length of 150 scans, and then decreases again for longer periods. Hence, taking into account the entire time series as it is usually done for conventional offline DCM analyses is suboptimal at least for our experimental task. This might reflect attentional fluctuations and fatigue, which are frequently observed in attention and other cognitive tasks (Weissman et al., 2006), but which are not taken into account by DCM. Because attentional modulation is often an experimental factor in DCM and because other modulating factors also vary over time, DCM might lead to better results when the time courses are sub-sampled with temporal windows over which the modulating factor is stable.

We deliberately did not optimize the model architecture, for example, by choosing the model that achieved the best performance based on the attention localizer run (which was similar to the neurofeedback runs). The purpose of rtDCM neurofeedback training is to modulate brain networks in ways that are defined by the experimenter for scientific or clinical reasons. Optimizing the model architecture might improve control over the feedback signal, but the optimal model based on localizer runs might (a) not be suitable to achieve the desired neurofeedback training effects (e.g. increasing top-down attention), and (b) might not be optimal in the neurofeedback situation. For example, in our study, the most promising models based on the attention localizer run were models with direct input into VC (Fig. 6, models 5–7), but we were primarily interested in providing feedback from a top-down attention model (Fig. 6; model 1). Moreover, the model architecture that we used during the neurofeedback runs performed poorly during the attention localizer run (Fig. 6; model 1), even though the experimental design and task were very similar between attention localizer and neurofeedback runs, i.e. they only differed in that the individual neurofeedback runs were longer, and in that the neurofeedback runs contained resting state as well as obviously feedback display conditions. Despite their similarity, our data show that visual–parietal connectivity during the neurofeedback runs cannot simply be predicted by connectivity patterns obtained during the attention localizer run. We can only speculate about the reasons why models with direct input into VC performed well during the attention localizer run, but showed the opposite pattern of results during the neurofeedback runs by significantly more often predicting dominance of M_aR_ during aL and dominance of M_aL_ during aR (Fig. 6). During the attention localizer run, activity in the VC did not change (Supplementary Methods and Inline Supplementary Table S3). However, during the neurofeedback runs, activity and CNR in both VCs decreased during the aL and the aR conditions (Table 2, Inline Supplementary Table S2). Moreover, it decreased more in the left compared to the right VC during aL, and vice versa during aR. Such a decrease of VC activity is surprising because previous studies found that attention increases activity in the contralateral visual cortex (Brefczynski and DeYoe, 1999; Hopfinger et al., 2000; Kastner et al., 1999; Lauritzen et al., 2009; Li et al., 2008; Silver et al., 2007).

Inline Supplementary Table S2

**Table S2:** CNRs in the ROIs during the neurofeedback runs.

condition	aL			aR		
run	1	2	3	1	2	3
**left SPL**	0.39±0.51	0.43±0.42	0.39±0.60	0.22±0.54	0.39±0.46	0.35±0.52
**right SPL**	0.17±0.51	0.06±0.54	0.12±0.50	0.05±0.59	-0.06±0.59	0.14±0.50
**left VC**	-0.61±0.49	-0.62±0.80	-0.62±0.78	-0.14±0.70	-0.12±0.84	-0.21±0.55
**right VC**	-0.13±0.54	-0.26±1.01	-0.35±0.78	-0.63±0.63	-0.49±0.67	-0.53±0.58

Inline Supplementary Table S2

Inline Supplementary Table S3

**Table S3:** Percent signal changes and CNRs in the ROIs during the attention localizer.

ROI / Cond	Percent		CNR	
aL	aR	aL	aR
**left SPL**	0.48±0.51	0.62±0.64	0.34±0.36	0.48±0.57
**right SPL**	0.28±0.32	0.33±0.51	0.28±0.27	0.23±0.44
**left VC**	-0.04±0.21	-0.04±0.34	-0.07±0.26	-0.04±0.39
**right VC**	-0.13±0.36	-0.06±0.48	-0.13±0.37	-0.14±0.46

Inline Supplementary Table S3

### Achieving control over the feedback signal

**Fig. S6:**
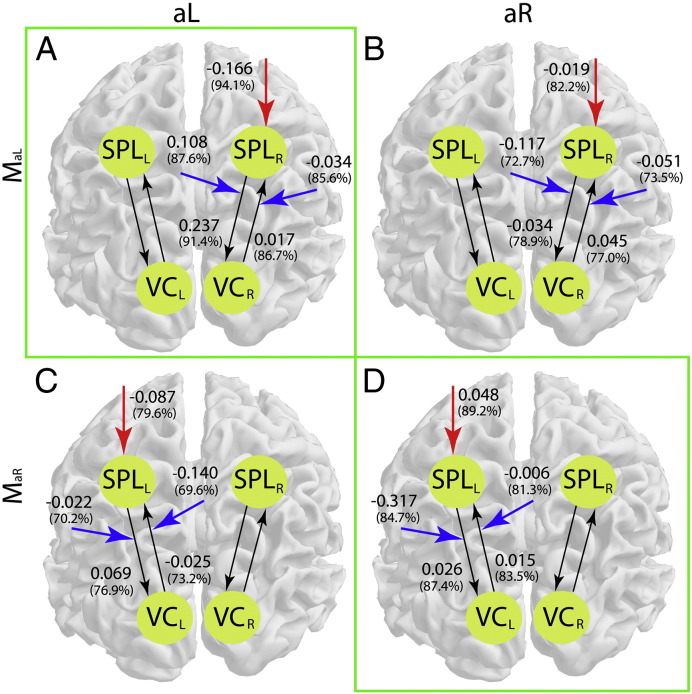
Model parameters of the successful trials. Parameters of M_aL_ are shown in the first row (A, B) and of M_aR_ are shown in the second row (C, D). The aL condition is shown in the first column (A, C) and the aR condition in the second column (B, D). As indicated by the green frame around A and D, M_aL_ was supposed to dominate over M_aR_ for the aL condition (model A vs. C), and M_aR_ was supposed to dominate over M_aL_ for the aR condition (model B vs. D). Model parameters are represented by the group mean value with their associated conditional probability in brackets. The parameter comparison between A and C as well as between B and D, which corresponded to our feedback signal, did not reveal any significant differences. Black arrows represent the connections. Red and blue arrows represent the external and modulatory inputs of attention, respectively. Note that the model parameters without attentional input were constant and therefore not shown. SPL — superior parietal lobule, VC — visual cortex, L — left hemisphere, R — right hemisphere.

In order to show the feasibility of rtDCM, we used a visual–spatial attention paradigm. It has been shown previously that shifting and maintaining visual–spatial attention can induce changes in connectivity between visual and parietal cortices that are detectable with DCM (Vossel et al., 2012). Also, visual–spatial attention is a cognitive factor that can easily be modulated by participants. Indeed, we found that participants were able to control the feedback signal by covertly shifting visual attention to the left during aL trials, and to the right during aR trials. The former led to dominance of M_aL_, and the latter to dominance of M_aR_ (Fig. 4). We did not find any significant DCM parameter changes that would help us to understand what model parameters underlie successful control of the connectivity feedback signal (Supplementary Methods and Inline Supplementary Fig. S6).

Inline Supplementary Figure S6

Even though there is no evidence for a statistically significant learning curve, voluntary control over the feedback signal improved across the neurofeedback runs and became statistically significant only in the last neurofeedback run (Inline Supplementary Fig. S3). However, our study was designed as a proof-of-principle study to show that the rtDCM-based feedback signal can be controlled, and not primarily to establish rtDCM-based neurofeedback learning. The relatively limited number of neurofeedback runs (i.e. 3 runs with a total of only 24 individual trials) was probably too short to determine significant learning effects. Also, we did not provide feedback from one specific model, but we assessed control over a feedback signal that involved dominance of one model in one condition and of another model in another condition, i.e. dominance of M_aL_ during aL and dominance of M_aR_ during aR. In addition, the sample size of 7 participants might have been too small to detect potential learning effects.

The shifts of attention that participants performed in order to control the feedback signal were also reflected in more conventional feedback measures based on brain activity. For example, differential feedback between the left and right visual cortices predicted the experimental condition, i.e. attention to the left or right (Inline Supplementary Fig. S5). This was expected because such activity changes have been reported to be associated with lateralized visual attention (Hopfinger et al., 2000; Kastner et al., 1999; Silver et al., 2007). However, there was no correlation between performance based on the differential feedback measures and performance based on rtDCM. This indicates that rtDCM provides a new and distinctive feedback measure; one that reflects connectivity between brain areas and that is qualitatively different from activity-based feedback from within a brain area.

### Future improvements of connectivity-based neurofeedback

Our study shows that connectivity-based neurofeedback is feasible. However, further improvements of our new approach will make neurofeedback training based on rtDCM more efficient. In general, performance levels and the delay of the feedback signal are very specific to the respective experimental paradigm. However, some key points likely affect most rtDCM paradigms. One of them is the choice of models and thus the ROIs that are being compared. For our specific paradigm, it is likely that a more careful choice or ROIs would have led to better control over the feedback signal. For example, the SPL ROI can be mapped more precisely (Silver et al., 2005), and the voxels in the visual ROI can be restricted to those voxels that show strong attentional modulation. It might also be beneficial to define all ROIs in a single localizer run so that they are based on the same cognitive process. Further, it is possible, that a visual target stimulus on which attention can be covertly shifted might improve the control over the feedback signal as well as reduce the time it takes to compute the feedback signal. Such a visual stimulus might make the task easier to perform, and it also adds another direct input variable to the DCM that might facilitate model convergence and stability. In the present experiment, we did not include visual stimulation because we focused on a top-down attention model that eventually allows for transferring the learned control to a situation outside the fMRI scanner where there is no specific visual stimulation.

Besides factors related to the experimental paradigm, several other, more general factors will make rtDCM more efficient. For example, higher field strengths in principle increase the fMRI contrast-to-noise ratio and thus may increase control over the rtDCM feedback signal as well as computational speed. In addition, increased computational power will help to further reduce the time it takes to compute the rtDCM feedback signal (Moore, 1965).

## Conclusions

Neurofeedback studies based on rtfMRI so far have been limited by training activity only within a single brain area. However, most mental functions and most neurological disorders are associated with changes in connectivity between brain areas. The present study is the first report of successful connectivity feedback based on rtfMRI. This important extension of the neurofeedback approach allows to non-invasively and non-pharmacologically manipulate brain connectivity directly. It will thus contribute to the development of neurofeedback as a promising research tool and will lay the foundations for important clinical applications.

The following is the supplementary data related to this article.

Supplementary methods

## Figures and Tables

**Table 1:**
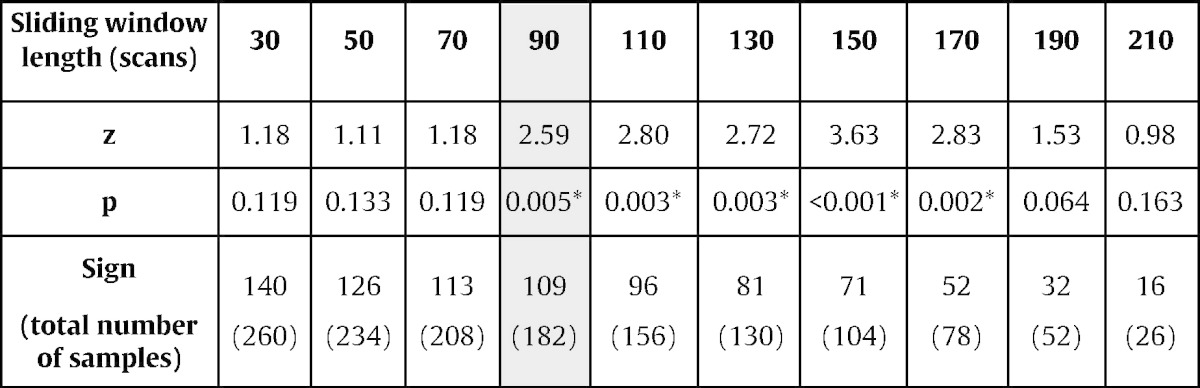
Optimization of the sliding window. For each sliding window length, we determined how often the real-time Bayesian model comparison reflected the respective experimental condition of the localizer runs, i.e. how often M_aL_ was dominant during aL conditions and vice versa for M_aR_ and the aR condition. The shortest possible sliding window comprised 90 scans, the best one 150 scans, and performance surprisingly decreases for longer sliding windows. For the neurofeedback runs, we used a sliding window of 90 scans (highlighted in gray). Z represents the z-score, *p* indicates significance of the applied sign test, and *sign* represents the number of positive *samples* based on the sign test. Asterisks denote statistical significance.

**Table 2:** Percent signal changes in the ROIs during the neurofeedback runs.

Condition	aL			aR		
Run	1	2	3	1	2	3
Left SPL	0.22 ± 0.47	0.47 ± 0.46	0.35 ± 0.40	0.13 ± 0.47	0.46 ± 0.42	0.37 ± 0.48
Right SPL	0.05 ± 0.54	0.18 ± 0.53	0.11 ± 0.39	0.0 ± 0.51	− 0.02 ± 0.50	− 0.02 ± 0.64
Left VC	− 0.60 ± 0.75	− 0.67 ± 1.10	− 0.60 ± 0.69	− 0.15 ± 0.40	− 0.22 ± 0.56	− 0.33 ± 0.54
Right VC	− 0.28 ± 0.55	− 0.18 ± 0.92	− 0.40 ± 0.77	− 0.58 ± 0.63	− 0.44 ± 0.86	− 0.47 ± 0.63
