# Supplementary material for: Connectivity-based neurofeedback: Dynamic causal modeling for real-time fMRI
Source: Neuroimage. 2013 Nov 1;81:422–30. doi: 10.1016/j.neuroimage.2013.05.010 (PMC3734349; doi:10.1016/j.neuroimage.2013.05.010)
Supplement: Inline Supplementary Table S1 [file mmc1.docx]

| **run/**  **parameter** | | **localizer** | | **neurofeedback** | |
| --- | --- | --- | --- | --- | --- |
|  |  | **condition** | **baseline** | **condition** | **baseline** |
| **Heart rate** | | 66.0±10.8 | 66.5±10.5 | 67.6±8.3 | 67.1±8.0 |
| **Respiration** | | 60.0±18.3 | 59.4±18.6 | 59.3±18.1 | 58.7±18.4 |
| **Eye movement** | **h** | 1.58±0.73 | 1.51±0.74 | 1.48±0.71 | 1.39±0.67 |
|  | **v** | 1.66±0.91 | 1.52±0.74 | 1.64±0.91 | 1.54±0.82 |

**Supplementary Table 1.** Heart rate [BPM], respiration [a.u.], horizontal and vertical eye movements from the center [°].
