# Supplementary material for: Connectivity-based neurofeedback: Dynamic causal modeling for real-time fMRI
Source: Neuroimage. 2013 Nov 1;81:422–30. doi: 10.1016/j.neuroimage.2013.05.010 (PMC3734349; doi:10.1016/j.neuroimage.2013.05.010)
Supplement: Inline Supplementary Table S2 [file mmc2.docx]

| **condition** | **aL** | | | **aR** | | |
| --- | --- | --- | --- | --- | --- | --- |
| **run** | 1 | 2 | 3 | 1 | 2 | 3 |
| **left SPL** | 0.39±0.51 | 0.43±0.42 | 0.39±0.60 | 0.22±0.54 | 0.39±0.46 | 0.35±0.52 |
| **right SPL** | 0.17±0.51 | 0.06±0.54 | 0.12±0.50 | 0.05±0.59 | -0.06±0.59 | 0.14±0.50 |
| **left VC** | -0.61±0.49 | -0.62±0.80 | -0.62±0.78 | -0.14±0.70 | -0.12±0.84 | -0.21±0.55 |
| **right VC** | -0.13±0.54 | -0.26±1.01 | -0.35±0.78 | -0.63±0.63 | -0.49±0.67 | -0.53±0.58 |

**Supplementary Table 2.** CNRs in the ROIs during the neurofeedback runs.
