# Supplementary material for: Connectivity-based neurofeedback: Dynamic causal modeling for real-time fMRI
Source: Neuroimage. 2013 Nov 1;81:422–30. doi: 10.1016/j.neuroimage.2013.05.010 (PMC3734349; doi:10.1016/j.neuroimage.2013.05.010)
Supplement: Inline Supplementary Table S3 [file mmc3.docx]

| **ROI / Cond** | **Percent** | | **CNR** | |
| --- | --- | --- | --- | --- |
|  | **aL** | **aR** | **aL** | **aR** |
| **left SPL** | 0.48±0.51 | 0.62±0.64 | 0.34±0.36 | 0.48±0.57 |
| **right SPL** | 0.28±0.32 | 0.33±0.51 | 0.28±0.27 | 0.23±0.44 |
| **left VC** | -0.04±0.21 | -0.04±0.34 | -0.07±0.26 | -0.04±0.39 |
| **right VC** | -0.13±0.36 | -0.06±0.48 | -0.13±0.37 | -0.14±0.46 |

**Supplementary Table 3.** Percent signal changes and CNRs in the ROIs during the attention localizer.
