# Supplementary material for: Connectivity-based neurofeedback: Dynamic causal modeling for real-time fMRI
Source: Neuroimage. 2013 Nov 1;81:422–30. doi: 10.1016/j.neuroimage.2013.05.010 (PMC3734349; doi:10.1016/j.neuroimage.2013.05.010)
Supplement: Supplementary methods [file mmc4.pdf]

Supplementary Tables

[insert Supplementary Table 1 about here]

[insert Supplementary Table 2 about here]

[insert Supplementary Table 3 about here]

Supplementary Figures

[insert Supplementary Figure 1 about here]

[insert Supplementary Figure 2 about here]

[insert Supplementary Figure 3 about here]

[insert Supplementary Figure 4 about here]

[insert Supplementary Figure 5 about here]

[insert Supplementary Figure 6 about here]
